# Exploring the open-circuit voltage of organic solar cells under low temperature

**DOI:** 10.1038/srep11363

**Published:** 2015-06-16

**Authors:** Boyuan Qi, Qing Zhou, Jizheng Wang

**Affiliations:** 1 Beijing National Laboratory for Molecular Sciences, Key Laboratory of Organic Solids, Institute of Chemistry, Chinese Academy of Sciences, Beijing 100190, P.R. China; 2 Graduate University of Chinese Academy of Sciences, Beijing 100049, P. R. China

## Abstract

Open-circuit voltage (V_OC_) in organic solar cells (OSCs) is currently still not well-understood. A generally acceptable view is that V_OC_ is mainly determined by the energy level offset between donor and acceptor materials. Recently in ternary blend OSCs, V_OC_ is found to be dependent on the blend composition. But contrary to expectation, this dependence is not a simple linear relationship, which adds complications to understanding on V_OC_. Here, in order to figure out the origin of V_OC_, we performed a series of experiments on both binary and ternary blend OSCs in a wide temperature range from 15 K to 300 K. It is observed that the devices behave like Schottky barrier (SB) diode. By fitting the experimental results with SB diode model, the detailed device parameters of ternary blend OSCs are extracted and it is found that V_OC_ is determined by the energetics of organic molecules and metal at the cathode interface, and the inhomogeneity of the SB also play a great role in the origin of V_OC_ at low temperatures. This work not only paves the way to deep understanding on the origin of V_OC_, but also opens a door to further exploring the general working principle of OSCs.

Pursuing higher power conversion efficiency (PCE) has always been a striving direction for researchers to achieve commercialization of the organic solar cells (OSCs). However, it is found that between the two parameters that determining PCE of OSCs, namely, open-circuit voltage (V_OC_) and short-circuit current density (J_SC_), there is a trade-off. Furthermore, the origin of V_OC_ is not completely clear, which remains a challenge for better understanding on OSCs and for its further optimization. Recently, OSCs based on ternary blend have been found to have unique properties and evoke some interesting areas in this direction^1^,^2^,^3^,^4^,^5^,^6^,^7^,^8^,^9^,^10^. Ternary blend OSCs composed of a fullerene acceptor and two polymeric donors with complementary absorption can have obvious enhancement in the J_SC_, however it is also found that the V_OC_ changes with the blend composition in these devices. Ternary blends with appropriate composition can achieve PCE exceeding those of each devices based on their corresponding binary blends^2^,^3^,^6^,^8^. Actually, the characteristics of tunable V_OC_ appear in almost all the ternary blend OSCs, both two donors mixed with one acceptor and two acceptors with one donor^1^,^2^,^3^,^4^,^5^. In conventional binary blend devices, V_OC_ is determined by the energy level offset between donor and acceptor material, which is expressed by the following equation^11^:


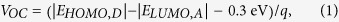


where q is the elementary change, *E*_*HOMO,D*_ is the highest occupied molecular orbital (HOMO) level of donor, and *E*_*LUMO,A*_ is the lowest unoccupied molecular orbital (LUMO) level of acceptor, the 0.3 V loss of V_OC_ is just an empirical value, it may be greater or smaller in different systems. The 0.3 V loss is expected to have two reasons, one is the tail states induced by the disorder in blend, the other is the energy loss induced by carriers’ recombination^12^. Here if it is assumed that ternary blend OSCs also obey this equation, and the equivalent HOMO (LUMO) level of two donors (acceptors) is their weighted average value, then the V_OC_ will show a linear dependence on the amount of the two donors (acceptors) in blends. Interestingly, in most practical experiments, the V_OC_ dependence on composition is not linear. To explain this observation, Li and co-workers proposed a three-diode model^7^. In this model ternary blend is regarded as the mixture of their two corresponding binary blends, each of which could be represented by a conventional diode. Under light, the two diodes can provide current to the outside overloads separately. However the two acceptors have different LUMO levels, and electrons prefer to flowing from high energy level to low energy level, so there should be another current leakage from one diode to the other. This unidirectional current is depicted by a third diode in the three-diodes model, and it is just the reason that variation of V_OC_ with blend composition is not linear. Duck *et al.* proposed the similar explanation with a parallel circuit model^4^, in which ternary blend has also been regarded as the combination of two binary blends, while they thought that the non-linear dependence of V_OC_ on composition is influenced by the bulk components of the series resistance. Street *et al.* have pushed this work a step further, they confirmed the continuous variation in energy of the HOMO (LUMO) level of two donor (acceptor) materials in the blend by using photocurrent spectral response (PSR) to measure the electronic states of ternary blend. They still observed that the optically excited exciton states do not reflect the average composition, but retain individual molecular characteristics, which indicates the formation of alloy^5^.

In order to get further insight into the origin of V_OC_ in OSCs, in this paper we carry out a series of experiments on the devices based on both binary and ternary blend, under a wide range of temperature from 15 K to the room temperature (300 K). It is observed that, the ideality factor, which is a sign of the diode property shows a steep increase at low temperature. This observation is in agreement with results obtained in inorganic Schottky barrier (SB) diode devices^13^,^14^,^15^,^16^. This finding strengthens the idea proposed in OSCs that the SB at the cathode interface plays a major role in the origin of V_OC_^17^. Then analyses are performed on the current density-voltage (J-V) characteristics with the thermionic emission (TE) theory from inorganic SB diodes, and SB heights are calculated for the devices with different blend composition. These results show that V_OC_ is mainly determined by the SB formed at the cathode interface and influenced by the surface states and inhomogeneity of SB at the interface.

## Results

### J-V characterization

**Figure 1:**
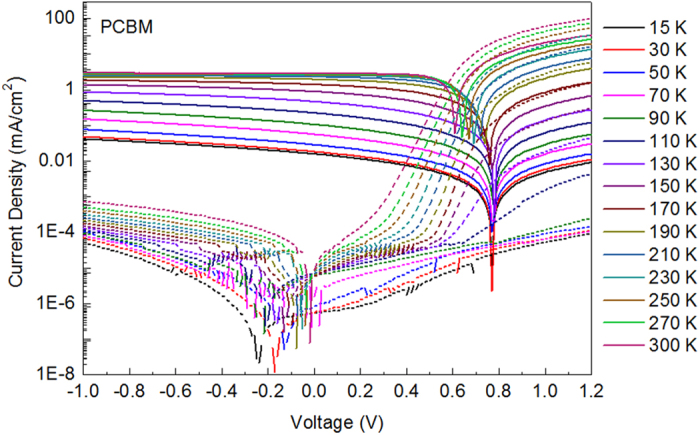
Device performance under illumination and in dark. J-V characteristics of PCBM device measured in the temperature range of 15–300 K.

**Figure 2:**
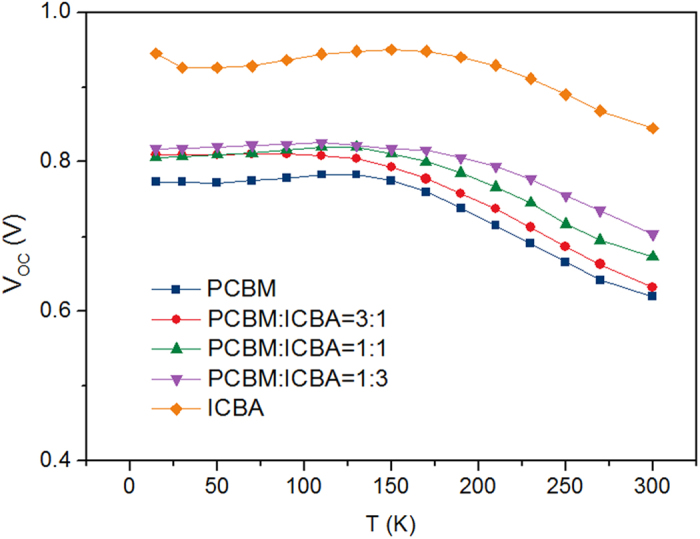
V_OC_ comparison. V_OC_ values for PCBM, 3:1, 1:1, 1:3 and ICBA devices under different temperatures.

The structure of the OSC devices is indium tin oxide (ITO)/poly(3,4-ethylenedioxythiophene):poly(styrene sulfonic acid) (PEDOT:PSS)/ blend/Ca/Al. Here the blend consists of one donor material, poly(3-hexylthiophene) (P3HT) and two acceptor materials, namely, phenyl-C_61_-butyric acid methyl ester (PCBM) and indene-C_60_ bisadduct (ICBA). To facilitate the comparison of device parameters, we prepare the solution of blend with the ratio as P3HT:PCBM:ICBA = 20:20:0 mg/ml, 20:15:5 mg/ml, 20:10:10 mg/ml, 20:5:15 mg/ml, 20:0:20 mg/ml, respectively, and the concentrations of both overall solute and P3HT are kept unaltered. Then all the solutions are spin-coated at the same speed and thermally heated at 110 °C, in order to keep the thicknesses of these active layers similar to each other. Here, the thicknesses of the optimized active layers are all around 150 nm. In the following, the five kinds of devices are denoted by PCBM, 3:1, 1:1, 1:3, and ICBA device for convenience. After evaporation of electrodes, devices are transferred from glove box into the vacuum chamber for low-temperature measurements. With the closed cycle refrigerator, the temperature of the device could be reduced to as low as 15 K. Then the J-V and (capacitance-voltage) C-V measurements are performed in the range of 15 ~ 300 K, with a step of 20 K. The light and dark J-V curves of PCBM device are shown in Fig. 1. (J-V curves of other four devices are shown in Supplementary Figure S1.) All the parameters for the five devices extracted in J-V measurements are listed in Supplementary Table S1, it should be noted here that these measurements are performed under AM 1.5G illumination of 0.8 suns intensity, so the J_SC_s are lower than the values in standard tests. From the J-V curves in dark, two parameters characterizing the property of diodes, namely ideality factor (n) and saturation dark current density (J_0_), can be extracted with Shockley equation listed below (details can be found in the supporting information of ref. 18):


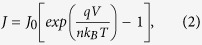


where q is the elementary charge, *k*_*B*_*T* the thermal energy. In order to facilitate comparison, the extracted parameters under different temperature are put together, which are shown in Fig. 2 and Supplementary Figure S2. In Fig. 2, it can be seen that dependencies of V_OC_ on temperature for the five devices show the same trend: V_OC_ increases first when temperature decreases until about 150 K, V_OC_ begins to saturate and then drops a little. This trend has also been observed by other groups^19^,^20^. They attributed the nonlinear dependence of V_OC_ on T to the low mobility at low temperature, and as a result, the photogenerated carriers are localized and V_OC_ is reduced^20^. In Figure S2(a), (b) and (d), it is shown that J_SC_ and FF decrease with decreasing T, and R_s_ increases obviously with decreasing T. These all imply that the mobility of charge carriers at low temperature are very small and carriers can not be extracted by the electrodes efficiently. However, it is worth noting here that n and J_0_ in Figure S2(f), (g) show strange trend with T. Generally, n is in the range of 1 ~ 2, which reflects the mechanism of current flowing inside the device. When n = 1, diffusion current dominates in the device, while recombination current dominates when n = 2. However, in Figure S2(f) it can be seen that the values of n for these devices exceed 2 and increase exponentially when T is lower than 190 K. J_0_, meanwhile, increases inversely with T. From the reverse bias region of the dark current curves in Fig. 1, it can be seen that the reverse dark current increases with T in the whole temperature range of 15 ~ 300 K. We hypothesize that this contradiction may be caused by the R_s_. Because at low temperature, R_s_ becomes larger and larger, which is comparable to R_sh_, and hence more voltage drops on R_s_ and the Shockley equation can not be simplified into the form of equation (2). Here in order to test this hypothesis, we re-extract n and J_0_ with the Shockley equation which takes both R_s_ and R_sh_ into consideration:


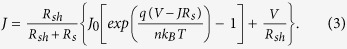


The re-extracted n and J_0_ are shown in Supplementary Figure S3. Interestingly, it can be seen that in relatively high temperature range (190–300 K), the values for n are approximately equal. While at low temperature, although the re-extracted n is smaller than its previous value, it is still larger than 2 and the changing trend that n increases exponentially with T still remains unchanged. Based on these observations, we suppose that at low temperature there must be some other factors that can not be demonstrated by the Shockley equation.

### Schottky Barrier Diode

In inorganic SB diodes, the observations that the ideality factor increases inversely with T at low temperature are widely reported. It is attributed to the presence of SB inhomegeneity^13^,^16^ at the metal/semiconductor (M/S) interface, i.e., there is a spatial distribution of Schottky barrier patches. Since the current transport across the SB is a process dominated by the temperature, the charge carriers at low temperature prefer to going through the SB patches with lower energy, which finally leads to a larger ideality factor^13^,^16^.

Here in order to figure out the dependence of V_OC_ on the composition in ternary blend OSCs, we also introduce the SB diode model in this work. Actually, this model has also been used in OSCs by other groups, and has been proven to fit well with the experimental results^21^,^22^. In this model, the blend of donor and acceptor is regarded as one p-type semiconductor material (because the conjugated polymer is usually p-doped when exposed to air or moisture). Therefore the Fermi level of the blend is in the vicinity of HOMO level of the donor material. When the blend contacts with the electrodes, at the anode side, the Fermi level of blend is close to the work function of PEDOT:PSS, so Ohmic contact forms at this side; while at cathode side, because the Fermi level difference between blend and electrode (Ca, Al) is large, band bending occurs and SB forms at this side. The height of the SB (Φ_*B*_) is determined by the Fermi level of cathode (Φ_*c*_) and HOMO level of the blend (*E*_*HOMO*_) at the interface of SB:


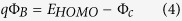


Here in order to calculate the barrier height at the cathode, we use the TE theory which is usually used to predict the J-V characteristics in the device under applied bias^13^,^16^:


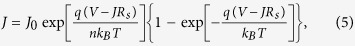


here J_0_ is the reverse saturation dark current density which can be expressed as


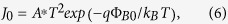


where A^*^ is the effective Richardson constant, Φ_*B0*_ is the zero-bias barrier height of the Schottky diode. By taking the natural logarithm of equation (6), it can be rewritten as


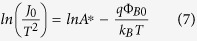


**Figure 3:**
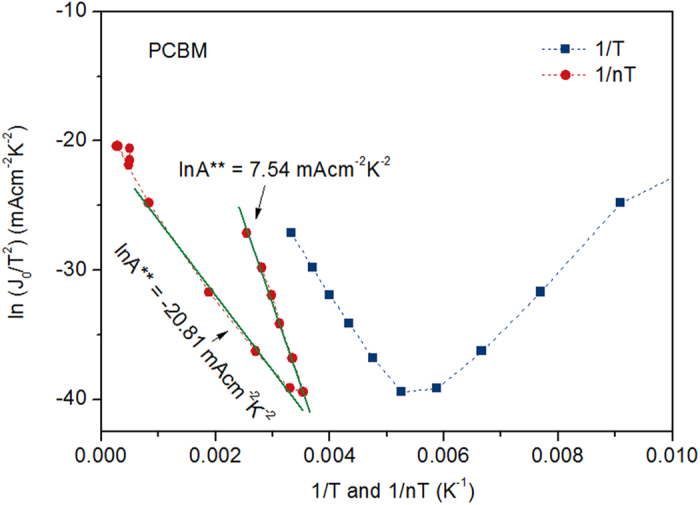
ln(*J_0_/T^2^*) vs 1/T and 1/nT. The plots of *ln*(*J*_0_/*T*^2^) with 1/T and 1/nT for PCBM device.

Equation (7) predicts that if *ln*(J_0_/*T*^2^) is plotted with 1/T, the plot will be a linear line, whose slope determines the Φ_*B*0_ and intercept gives the Richardson constant. Figure 3 shows the plot of *ln*(J_0_/*T*^2^) with 1/T, from which it can be seen that at high temperature, the experimental data can be fitted with a linear line. When temperature is low, however, the slope of the plot changes from negative to positive values, which implies a negative Φ_*B*0_. This could be explained by the strong dependence of Φ_*B*0_ and n on temperature. Similar results have been reported in inorganic SB diode^13^,^16^, and it has also been found that if *ln*(J_0_/*T*^2^) plotted with 1/nT, the curve is more linear than *ln*(J_0_/*T*^2^) vs 1/T. Here we also plot *ln*(J_0_/*T*^2^) with 1/nT in Fig. 3 (plots for other four devices are shown in Supplementary Figure S4). It can be seen that the plot has two linear parts with different slopes. Also noticeable is that at low temperature (T < 190 K), the slope of the curve shows a gradual and small change with decreasing temperature. It has been reported that when operated at moderate temperatures, the current transport of SB diodes is dominated by the TE process. While when operated at low temperatures, tunneling current becomes more significant in the SB diodes. Then current density could be expressed as below with tunneling current taken into account^23^:


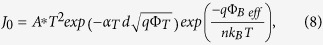


where 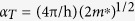 is a constant that depends on the tunneling effective mass and Planck’s constant h, Φ_*T*_ is the effective barrier height for tunneling, and width of the barrier is d, Φ_*B*0_ is replaced with Φ_*B eff*_/*n*. Equation (8) is the combination of TE equation with the tunneling probability 
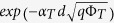
. By taking the natural logarithm of equation (8), it can be rewritten as


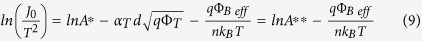


**Figure 4:**
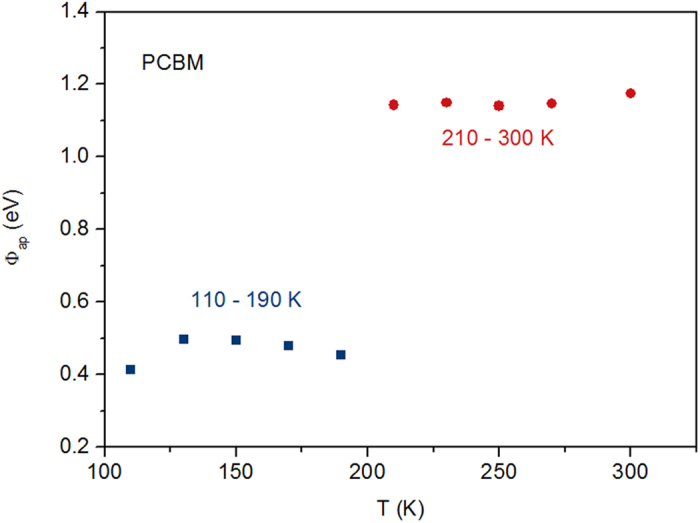
Apparent SB height. The calculated apparent SB height under different temperatures of PCBM device.

Here 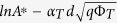 is substituted with lnA^**^ for simplicity, which can be calculated by the intercept of plots with y axis. As mentioned above, at high temperatures (about 210–300 K), the current transport is dominated by the TE process, hence in this case, 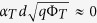 and lnA^**^ ≈ lnA^*^. While at low temperatures (T < 190 K), tunneling current gains more significance and lnA^**^ < lnA^*^. And at the interface of cathode, there exists barrier inhomogeneity. With the decreasing of temperature, more patches of SB with low height participate in the conduction of current, and the average barrier height for tunneling will become lower and lower. Accordingly, 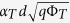 becomes less and lnA^**^ increases. This is the reason why slope of *ln*(*J*_0_/*T*^ 2^) with 1/nT plot decreases gradually at low temperature. Here we assume lnA^**^ to be a constant (by fitting the plot with a linear line) at low temperature for convenience. By bringing lnA^**^ back into equation (9), Φ_*B eff*_ can be obtained and Φ_*B*0_ could be calculated by Φ_*B eff*_ /*n*, which are shown in Fig. 4. It could be seen that the barrier heights split into two groups, between which there is an abrupt break. This is because we use a constant lnA^**^ roughly for calculation, rather than gradually changing lnA^**^ in the low temperature range. Actually Φ_*B*0_ should vary continuously in the whole temperature range if lnA^**^ is calculated accurately.

Generally the distribution of SB energy levels are depicted with the Gaussian distribution^24^,^25^,^26^,^27^:


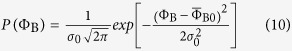


The overall current density across the interface is calculated by integrating the current transporting through the SBs with different barrier heights:


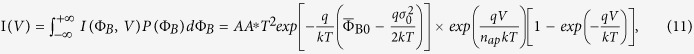


where *n*_*ap*_ is the apparent ideality factor, and the Gaussian distribution of apparent barrier height could be represented by:


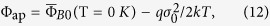


where σ_0_ is the zero bias standard deviation of the SB height distribution and it is a measure of the barrier homogeneity. The temperature dependence of σ_0_ is usually small and can be neglected, so Gaussian distribution do not change with the temperature. It can be imagined that at low temperature the density of carriers is small, the current tends to flow though lower SBs, so the apparent SB height is low; when temperature increases, the density of carriers will increase with temperature, however the amount of lower SBs is limited, so some current begins to flow accross higher SBs, as a result, the apparent SB height increases.

**Figure 5:**
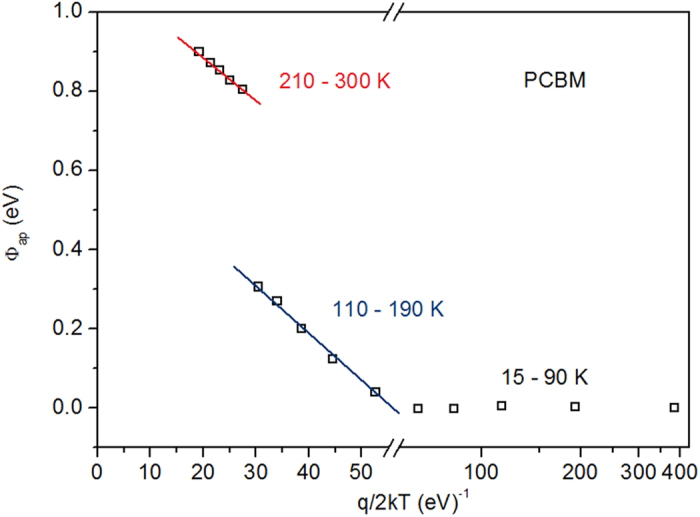
Distribution of apparent SB height with Gaussian model. The apparent SB height plotted with q/2kT, assuming the Gaussian distribution of the spatial barrier heights.

From equation (12), the average barrier height and barrier homogeneity could be quantified. The results for PCBM device are shown in Fig. 5. By extending the fitting line to the y axis, the average barrier height for the SB at high temperature and low temperature could be calculated (It should be noted here that the apparent barrier heights at low temperatures are approximate values because they are calculated with an approximate lnA^**^). For PCBM device, they are 1.102 eV (210–300 K) and 0.697 eV (110–190 K), respectively. From the slope of the fitting line, σ_0_ of device in high temperature range is 0.110 V. Φ_*ap*_ vs q/kT plots of other four devices are shown in Supplementary Figure S5 and the calculated average Φ_*B*0_ at T = 0 K is listed in Table 1. From Table 1 we can see that PCBM and ICBA devices have the least and largest SB height, respectively. And devices with different ratio of acceptors have the median values of SB height. As we know, V_OC_ is determined by the dark current in the device^12^,^28^ (J_SC_s for the five devices have similar values), while dark current is determined by the SB height, hence V_OC_ is finally determined by the SB height. From Table 1 it is found that the SB height of the five devices changes with the composition of the blend, and in consequence, V_OC_ changes with the composition of the blend.

Then the question arises here is that why SB height varies with blend composition. This may be attributed to the different cathode interface of PCBM and ICBA device^17^. Generally, disorder at the surface of the active layer induces additional energy levels into the band gap. What’s more, when cathode is thermally deposited onto the surface of the blend, metal diffusion and interaction with organic molecules may also lead to localized states at the surface^29^,^30^,^31^. In OSCs, these surface states locate at an interfacial layer formed by metal and organic molecules. This interfacial layer can exchange charge carriers with blend and cathode and withstand voltage drops on it. Then the built-in potential could be expressed as following^17^,^32^:


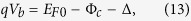


where Δ stands for the voltage drop across the interfacial layer. It has been reported that the fullerene energetics and donor: acceptor ratios play great roles in this process. Due to the constant P3HT amount in the five blends, different fullerene energetics of PCBM and ICBA could lead to different cathode interfaces, which may be the primary reason that the devices have different barrier heights.

**Figure 6:**
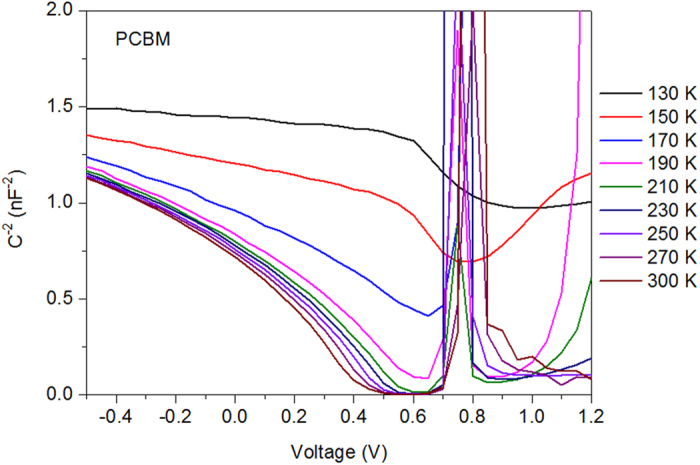
Built-in potential of PCBM device. Mott-Schottky plots of PCBM device under different temperatures.

**Figure 7:**
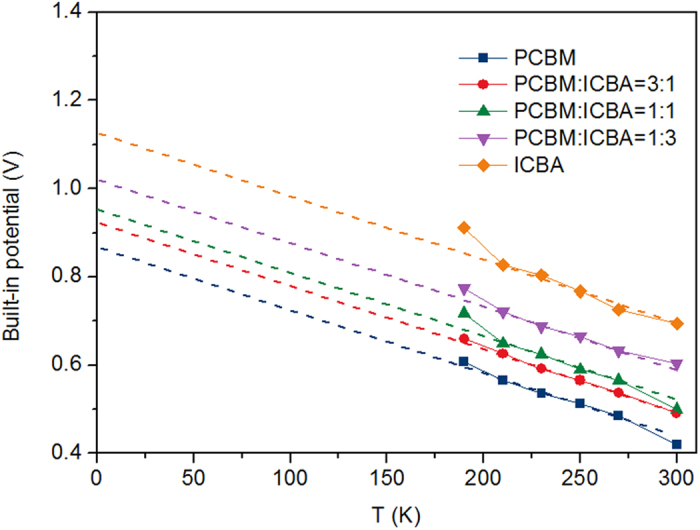
Built-in potentials comparison. Built-in potentials obtained by C-V measurements for PCBM, 3:1, 1:1, 1:3 and ICBA devices under different temperatures.

Here we measured the built-in potential by applying standard Mott-Schottky analysis to the results of C-V measurements which are performed with the electrochemical workstation. Figure 6 shows the C-V plots of PCBM device in the range from 130 K to 300 K. It can be seen that when T < 190 K, the capacitance can not response promptly with the AC signals and as a result, the curves are away from the x axis. This also enforces the previous analysis that that mobility of charge carriers at low temperature is low. Therefore the built-in potential calculated by Mott-Schottky analysis is not accurate when T < 190 K. The C-V plots of other four devices are shown in Supplementary Figure S6, where they also show similar tendencies. Figure 7 shows the calculated built-in potentials of PCBM, 3:1, 1:1, 1:3, and ICBA devices. It can be seen that the built-in potentials increase with the decreasing temperature. By fitting these values with linear lines, the built-in potentials at 0 K can be obtained from the extrapolated intersections of fitting lines with the y axis, which are listed in Table 1. As can be seen in Table 1, the built-in potential of ICBA device is larger than that of PCBM device, and the built-in potentials of 3:1, 1:1 and 1:3 devices are between the two. The Fermi levels of PCBM and ICBA devices are similar, hence according to equation (13) it is the different Δ that leads to different built-in potentials of PCBM and ICBA devices. Therefore the V_OC_ of ternary blend OSC is extremely dependent on the interfacial energetics. Different acceptor molecules (blended with donor molecules) interacted with metals directly produces an offset in the SB height. Due to the dark current originates from the carriers thermally activated across the SB, and dark current is the determining parameter for V_OC_, therefore V_OC_ is determined by the energetics at the cathode interface ultimately.

Finally we have also tried a different donor material with low band gap, thieno[3,4-b]-thiophene/benzodithiophene (PTB7), to study the applicability of SB model. Detailed results are shown in the Supplementary information, from which we can see that the PTB7 device behaves like the devices based on P3HT under low temperature. However what is interesting is that although PTB7:PCBM device has lower Φ_ap_ than that of P3HT:ICBA device under room temperature, its 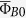 extracted at T = 0 K is larger than that of P3HT:ICBA device (shown in Table 1), i.e., the variation rate of Φ_ap_ with temperature in PTB7:PCBM device is larger than that of P3HT:ICBA device (which is obvious when comparing Supplementary Figure S5d with S10). In order to figure out this problem, we should view the energetics of the device. Generally in organic blend, the Fermi level could be expressed with:


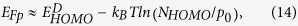


where *N*_*HOMO*_ is the density of states at the HOMO level, typical value of which is about 10^20 ^cm^−3^; *P*_0_ is the hole density caused by the doping, which can be calculated by the C-V measurement with Mott-Schottky analysis. In equation (4) Φ_*B*_ is determined by the difference of *E*_*Fp*_ and Φ_*c*_. Here P3HT:ICBA and PTB7:PCBM devices have the same Φ_*c*_, therefore Φ_*B*_ is finally determined by the *E*_*Fp*_. From equation (14), it can be seen that the variation rate of *E*_*Fp*_ depends on *ln*(*N*_*HOMO*_/*p*_0_). According to the C-V measurements, *P*_0_ in P3HT:ICBA and PTB7:PCBM devices are about 2.4 × 10^16 ^cm^−3^ and 1.1 × 10^16^ cm^−3^, respectively. As a result the variation rate of *E*_*Fp*_ with temperature in PTB7:PCBM device is larger than that of P3HT:ICBA device. This phenomena is also a strong evidence that the SB model applies to OSCs and the V_OC_ of OSCs originates from the cathode interface.

## Discussion

In conclusions, we have studied on the behaviour of both binary and ternary blend OSCs under low temperature. It is found that ideality factors of the devices show an obvious increase with the decreasing temperature. In order to explain this phenomenon, a model is introduced in which OSC is taken as SB diode. According to this model, the dark current in the device originates from the thermally emitted electrons across the SB. By fitting experimental data with the TE theory, it is observed that barrier inhomogeneity exists at the cathode interface. Especially under low temperature, electrons prefer to going across the SB patches with lower barrier height, and as a result, the ideality factors show increase under low temperature. We then calculate the barrier height of the devices and find that the barrier height changes with the blend composition, which shows the same trend with V_OC_ vs. blend composition. This is caused by the different cathode interface in PCBM and ICBA device, which is proved by the C-V measurements. At last we have also tried OSCs based on low band gap donor material, PTB7, and the experimental results show the validity of the SB model in low doping blends.

Our work gives a good explanation to the behaviour of OSCs in a wide range from 15 K to 300 K with the SB diode model, and uncovers the reason that the dependence of V_OC_ on blend composition in OSCs based on ternary blends. This finding proposes a new idea to gain further insight into the origin of V_OC_ in OSCs, and more importantly the general working principle of OSCs.

## Methods

### Device Fabrication

The pre-cleaned ITO glass substrate was firstly treated by oxygen plasma for 6 min, and then a 30 nm PEDOT:PSS (poly(3,4-ethylenedioxythio-phene):poly(styrenesulfonate), Clevios P VPAI 4083, from H. C. Starck) layer was spin-coated on the substrate, followed by thermal treatment at 140 °C for 10 min on a hotplate. The substrate was then transferred into the nitrogen glove box, and all the other processes were done in the glove box unless otherwise stated. P3HT:PCBM:ICBA were dissolved in o-dichlorobenzene (o-DCB) to be the active layer. Then the blend was spin-coated onto the substrate at 900 rpm for 15 s and covered in a glass Petri dish for solvent annealing. After that, all of the blends were annealed on a hot plate at 110 °C for 10 minutes to remove residual solvents. Finally, top electrodes were deposited in vacuum at a pressure of about 5.0 × 10^−5 ^Pa. The active device area was 4.4 mm^2^. In PTB7 device, in order to facilitate comparison with P3HT systems, PC_61_BM was chosen as the acceptor, the concentration of solution and process of spin coating also stay the same with those of P3HT:PCBM device. The difference is that PTB7:PCBM blend do not need solvent and thermal annealling, its solution was spin-coated onto the subtrate to dry.

### J-V Characterization

Current density-voltage (J-V) characteristics of the devices were measured with Keithley 4200 source meter and Newport 6279 NS solar simulator (450 W) with 80 mW/cm^2^ illumination. The measurements under low temperatures were performed in closed cycle refrigerator systems from Janis company, the cooling of samples were realized by providing high-pressure helium gas to the cold head with compressor.

### CV measurements

The capacitance-voltage (C-V) measurements were performed using a Zahner Zennium electrochemical workstation. They were recorded at a frequency of 1 kHz for extracting V_b_. The AC oscillating amplitudes were set as low as 10 mV (rms) to maintain the linearity of the response.

## Additional Information

**How to cite this article**: Qi, B. *et al.* Exploring the open-circuit voltage of organic solar cells under low temperature. *Sci. Rep.* **5**, 11363; doi: 10.1038/srep11363 (2015).

## Supplementary Material

Supplementary Information

## Figures and Tables

**Table 1:** Average SB heights and built-in potentials extracted at T = 0 K for PCBM, 3:1, 1:1, 1:3 and ICBA devices.

**Device**	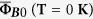 **[eV]**	***V***_***b***_** (T = 0 K) [V]**
PCBM	1.102	0.868
PCBM:ICBA = 3:1	1.118	0.923
PCBM:ICBA = 1:1	1.271	0.953
PCBM:ICBA = 1:3	1.336	1.021
ICBA	1.386	1.127

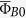
, the average zero-bias SB height; V_b_, built-in potential.
