# Supplementary material for: Exploring the open-circuit voltage of organic solar cells under low temperature
Source: Sci Rep. 2015 Jun 16;5:11363. doi: 10.1038/srep11363 (PMC4468816; doi:10.1038/srep11363)
Supplement: Supplementary Information [file srep11363-s1.pdf]

Supplementary Information

Exploring the open-circuit voltage of organic solar cells under low temperature

Boyuan Qi, Qing Zhou, and Jizheng Wang*

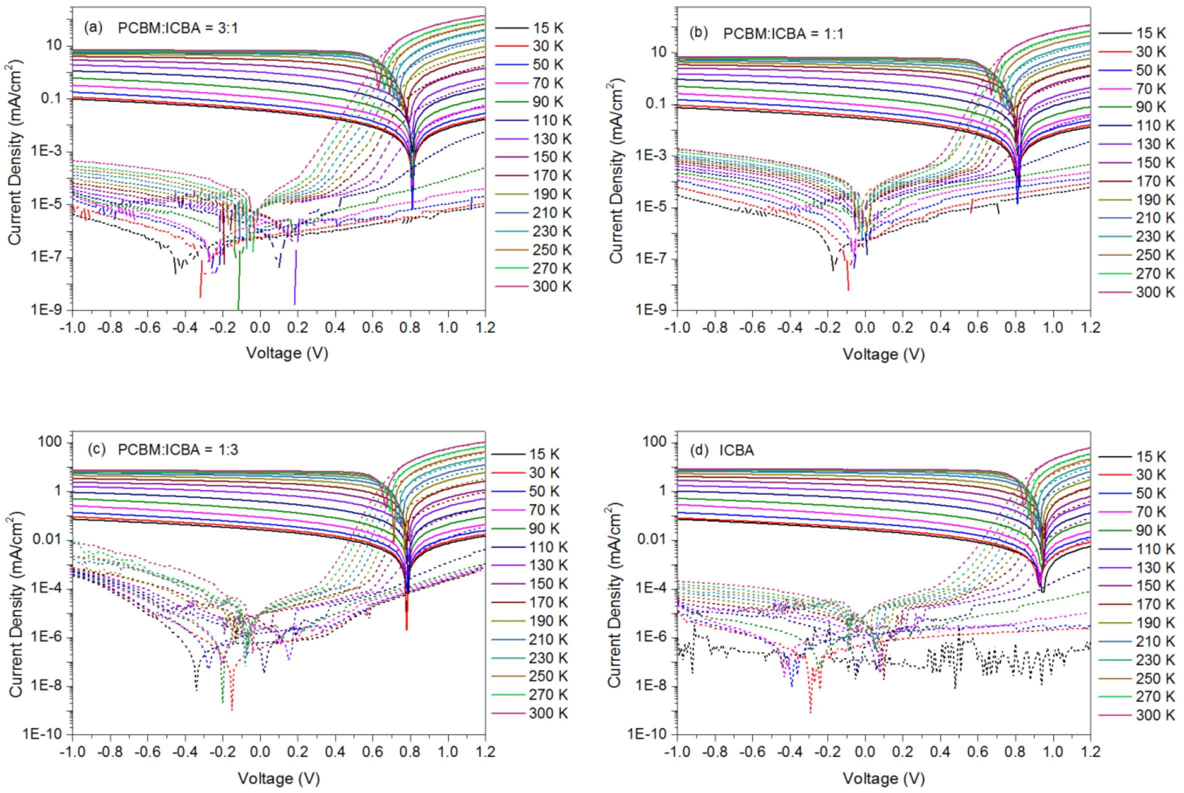

Figure S1 | Device performance under illumination and in dark. J-V characteristics measured in the temperature range of 15 - 300 K for (a) 3:1, (b) 1:1, (c) 1:3 and (d) ICBA devices.

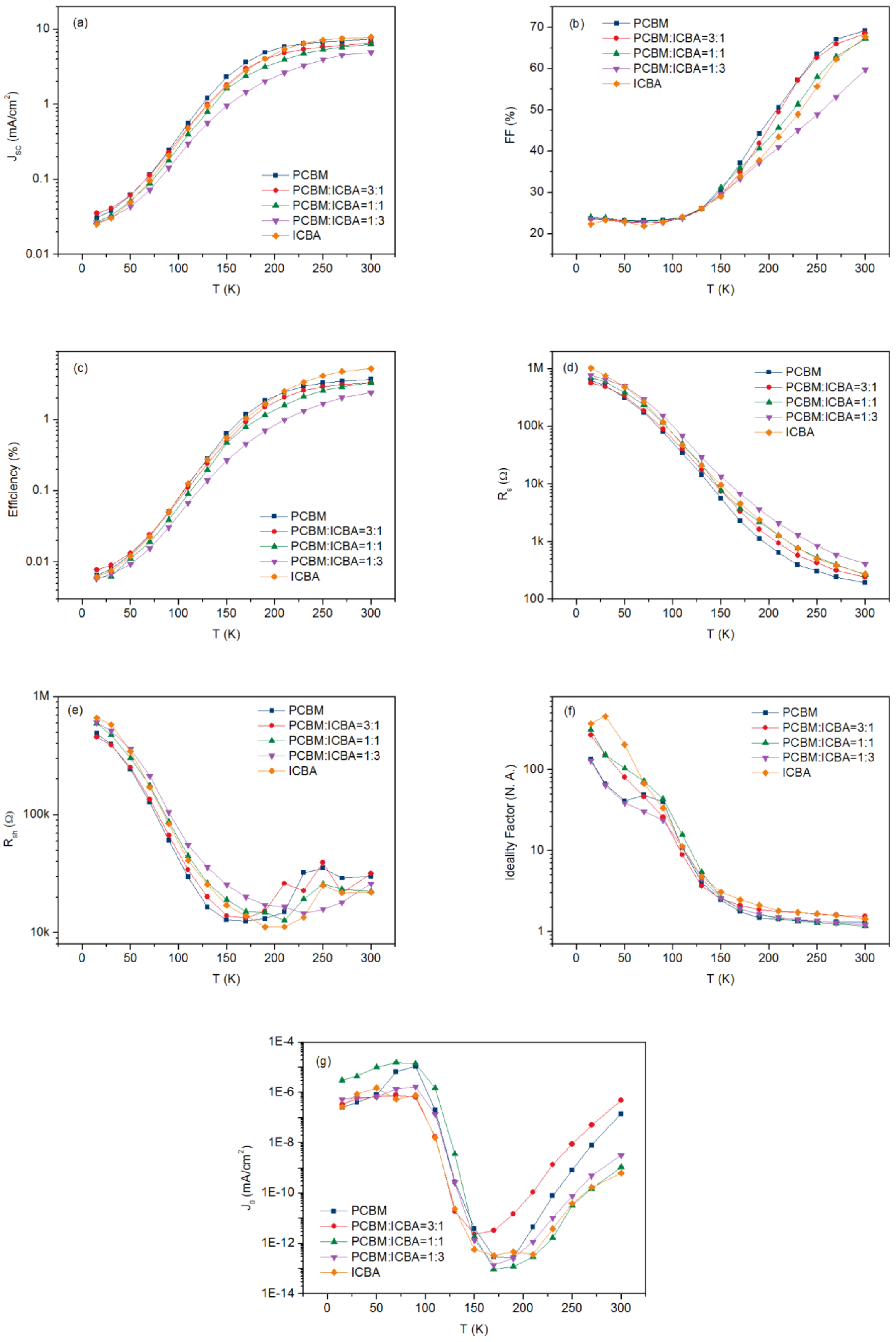

Figure S2 | Parameters comparison. Parameters extracted for PCBM, 3:1, 1:1, 1:3 and ICBA devices under different temperatures: (a) J_{SC}, (b) FF, (c) Efficiency, (d) R_s, (e) R_{sh}, (f) n, (g) J_0.

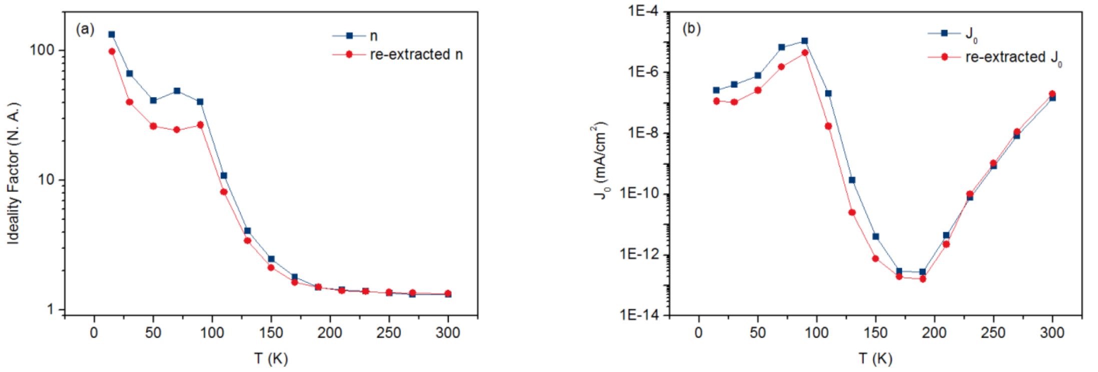

Figure S3 | Re-extracted parameters. Re-extracted n and J_0 with equation (3) for PCBM device.

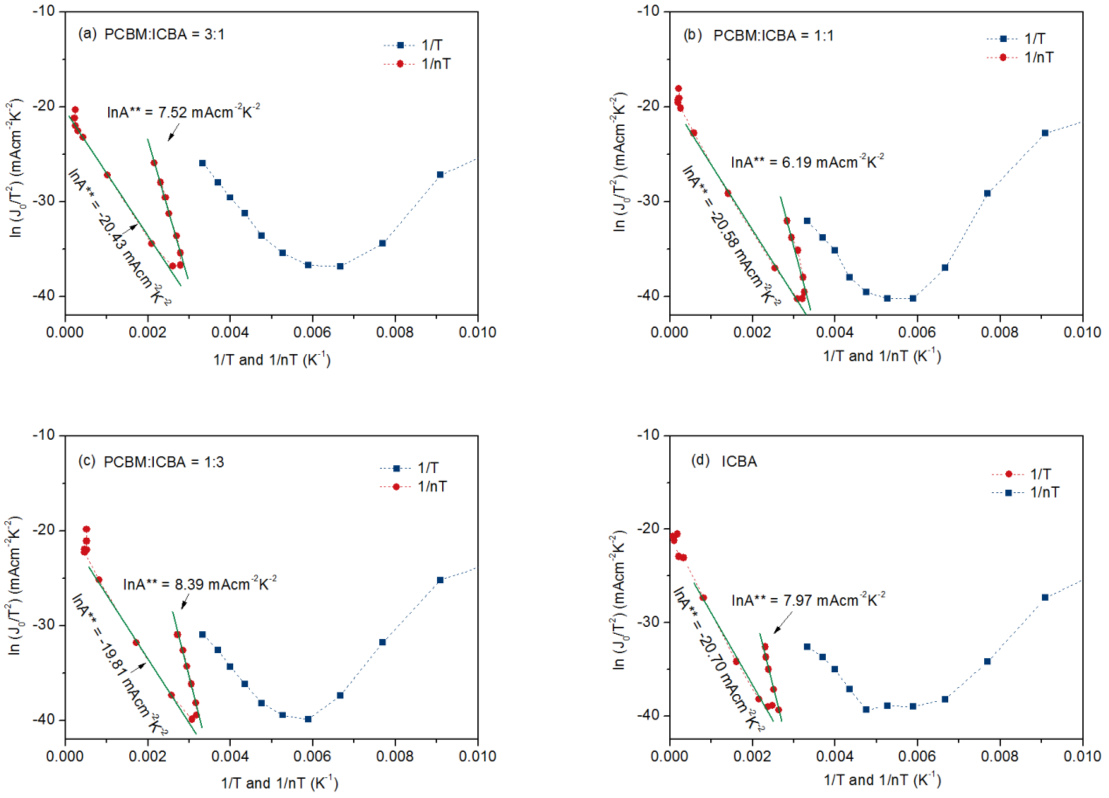

Figure S4 | $ln(J_0/T^2)$ vs 1/T and 1/nT. The plots of $ln(J_0/T^2)$ with 1/T and 1/nT for (a) 3:1, (b) 1:1, (c) 1:3, and (d) ICBA devices.

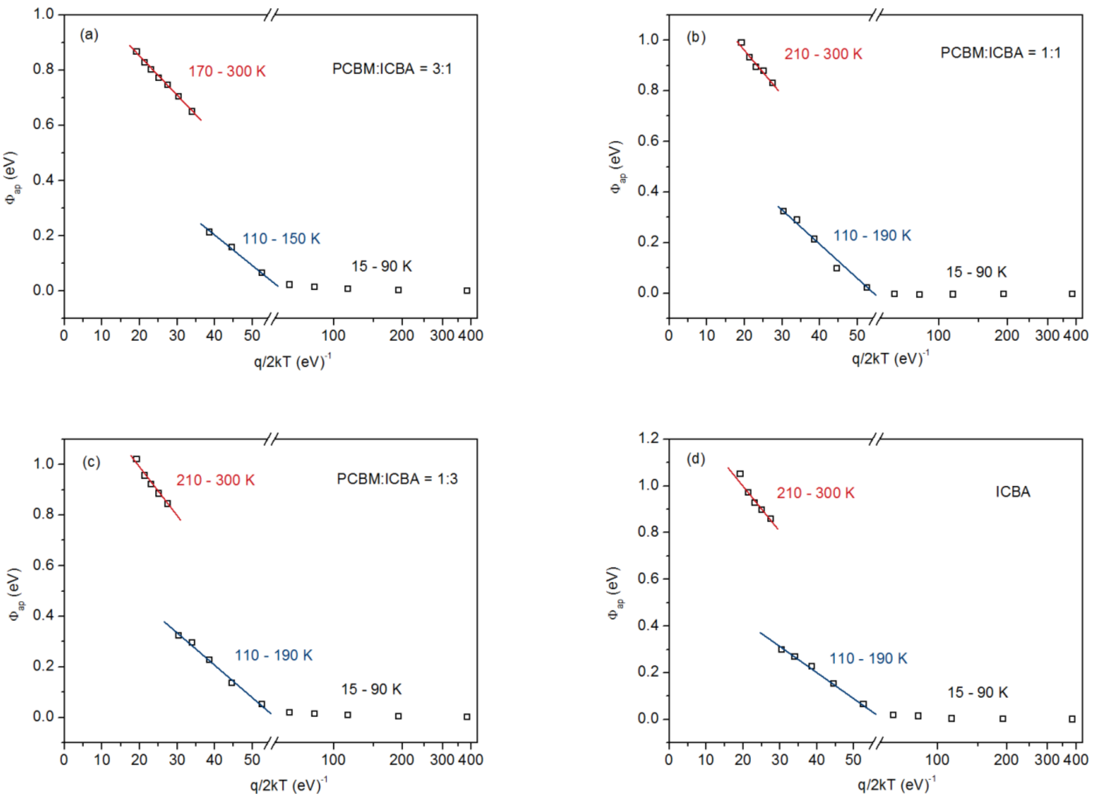

Figure S5 | Distribution of apparent SB height with Gaussian model. The apparent SB height plotted with q/2kT for (a) 3:1, (b) 1:1, (c) 1:3, and (d) ICBA devices.

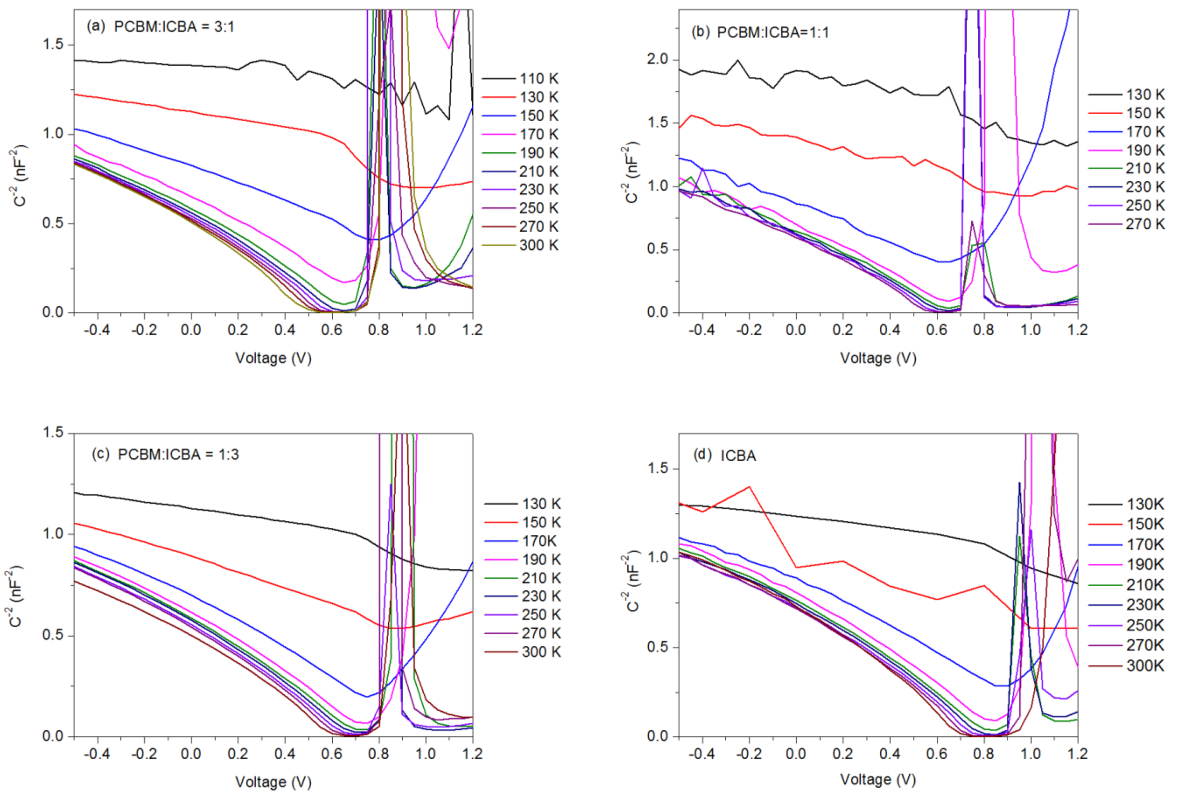

Figure S6 | Built-in potential. Mott-Schottky plots of (a) 3:1, (b) 1:1, (c) 1:3, and (d) ICBA devices under different temperatures.

Table S1 | Device performance and extracted parameters for PCBM, 3:1, 1:1, 1:3 and ICBA devices under different temperatures.

Device	T [K]	V_{OC} [mV]	J_{SC} [mA/cm^2]	FF [%]	PCE [%]	R_s [Ω]	R_{sh} [Ω]	n [n.a.]	J_0 [mA/cm^2]
PCBM	15	773	3.08E-2	23.7	6.50E-3	6.26E5	4.88E5	134	2.56E-7
	30	773	3.83E-2	23.6	8.00E-3	5.10E5	3.95E5	67.0	4.06E-7
	50	772	6.18E-2	23.2	1.28E-2	3.17E5	2.41E5	41.3	8.13E-7
	70	775	0.12	23.2	2.39 E-2	1.74E5	1.27E5	48.7	6.60E-6
	90	778	0.25	23.4	5.11 E-2	8.19E4	6.08E4	40.3	1.08E-5
	110	783	0.56	24.0	0.12	3.46E4	2.97E4	10.9	2.00E-7
	130	783	1.21	26.1	0.28	1.45E4	1.65E4	4.07	2.86E-10
	150	775	2.33	30.7	0.63	5.63E3	1.28E4	2.46	3.93E-12
	170	760	3.64	37.1	1.18	2.28E3	1.25E4	1.78	2.95E-13
	190	738	4.91	44.3	1.83	1.13E3	1.32E4	1.49	2.77E-13
	210	715	5.87	50.6	2.43	647	1.50E4	1.42	4.45E-12
	230	691	6.43	57.3	2.93	392	3.23E4	1.39	7.92E-11
	250	666	6.74	63.5	3.26	310	3.53E4	1.34	8.35E-10
	270	642	6.98	67.1	3.46	243	2.90E4	1.32	8.16E-9
	300	620	7.42	69.2	3.67	194	3.00E4	1.31	1.45E-7
PCBM:ICBA=3:1	15	810	3.54E-2	23.5	7.78E-3	5.66E5	4.55E5	269	3.28E-7
	30	810	4.12E-2	23.4	9.01E-3	4.90E5	3.91E5	153	5.58E-7
	50	810	6.18E-2	23.0	1.33E-2	3.36E5	2.52E5	81.1	6.73E-7
	70	810	0.11	22.7	2.40E-2	1.86E5	1.35E5	46.6	7.74E-7
	90	810	0.23	22.8	4.92E-2	9.06E4	6.70E4	25.9	6.65E-7
	110	808	0.50	23.8	0.11	4.08E4	3.42E4	8.91	1.83E-8
	130	804	1.00	25.9	0.24	1.77E4	2.02E4	3.68	1.88E-11
	150	793	1.83	29.5	0.49	7.54E3	1.39E4	2.56	2.32E-12
	170	777	2.98	34.9	0.93	3.39E3	1.34E4	2.11	3.31E-12
	190	757	4.06	41.9	1.49	1.64E3	1.52E4	1.89	1.49E-11
	210	737	4.86	49.5	2.04	943	2.62E4	1.77	1.10E-10
	230	713	5.43	57.2	2.55	576	2.27E4	1.73	1.39E-9
	250	687	5.82	62.7	2.89	430	3.93E4	1.65	8.92E-9
	270	663	6.06	66.0	3.06	317	2.18E4	1.60	5.11E-8
	300	632	6.61	68.5	3.30	243	3.18E4	1.55	4.83E-7
PCBM:ICBA=1:1	15	806	2.73E-2	24.1	6.07E-4	7.05E5	5.95E5	313	3.05E-6
	30	807	3.31E-2	23.9	6.34E-4	5.90E5	4.74E5	152	4.46E-6
	50	810	5.16E-2	23.2	1.12E-2	3.91E5	3.03E5	104	1.00E-5
	70	812	8.90E-2	23.1	1.92E-2	2.36E5	1.76E5	72.6	1.55E-5

	90	816	0.18	23.2	3.91E-2	1.16E5	8.77E4	43.6	1.42E-5
	110	820	0.40	23.9	0.09	4.96E4	4.49E4	15.7	1.52E-6
	130	820	0.79	26.1	0.20	2.17E4	2.63E4	5.48	3.68E-9
	150	811	1.62	31.2	0.47	7.72E3	1.90E4	2.62	1.91E-12
	170	800	2.39	35.9	0.79	3.82E3	1.50E4	1.90	9.56E-14
	190	785	3.16	40.6	1.16	2.21E3	1.49E4	1.64	1.22E-13
	210	766	3.96	45.7	1.58	1.28E3	1.27E4	1.46	2.97E-13
	230	745	4.76	51.4	2.09	767	1.93E4	1.35	1.68E-12
	250	717	5.37	58.0	2.55	536	2.58E4	1.29	3.34E-11
	270	695	5.74	63.0	2.88	398	2.34E4	1.26	1.53E-10
	300	673	6.32	67.3	3.28	271	2.25E4	1.18	1.08E-9
PCBM:ICBA=1:3	15	818	2.64E-2	23.6	5.82E-3	7.70E5	6.07E5	129	5.36E-7
	30	818	3.11E-2	23.4	6.80E-3	6.51E5	5.14E5	64.6	6.15E-7
	50	820	4.34E-2	22.7	9.31E-3	5.04E5	3.59E5	38.8	6.94E-7
	70	822	7.26E-2	22.8	1.56E-2	2.96E5	2.13E5	30.6	1.39E-6
	90	823	0.14	22.8	3.07E-2	1.55E5	1.05E5	23.8	1.70E-6
	110	826	0.30	23.8	6.70E-2	6.89E4	5.57E4	11.2	1.38E-7
	130	822	0.56	26.3	0.14	2.93E4	3.60E4	4.48	2.64E-10
	150	817	0.96	29.7	0.27	1.35E4	2.55E4	2.59	1.35E-12
	170	816	1.46	33.2	0.45	6.85E3	2.01E4	1.92	1.37E-13
	190	805	2.03	37.2	0.70	3.67E3	1.72E4	1.65	2.68E-13
	210	794	2.65	40.9	0.99	2.10E3	1.66E4	1.50	1.16E-12
	230	777	3.29	45.1	1.32	1.30E3	1.46E4	1.42	1.05E-11
	250	755	3.96	48.9	1.67	849	1.58E4	1.36	7.69E-11
	270	735	4.54	53.1	2.03	593	1.81E4	1.30	5.03E-10
	300	703	4.95	59.8	2.39	417	2.62E4	1.22	3.23E-9
ICBA	15	945	2.54E-2	22.4	6.20E-3	1.03E6	6.63E5	372	2.75E-7
	30	926	3.04E-2	23.3	7.54E-3	7.60E5	5.80E5	457	8.77E-7
	50	926	4.98E-2	23.0	1.21E-2	4.87E5	3.44E5	204	1.52E-6
	70	928	0.10	21.9	2.26E-2	2.69E5	1.71E5	67.6	5.47E-7
	90	936	0.21	22.9	5.10E-2	1.18E5	8.36E4	33.7	7.75E-7
	110	944	0.48	24.1	0.13	4.77E4	4.11E4	11.2	1.61E-8
	130	948	0.95	26.2	0.27	2.13E4	2.57E4	4.79	2.43E-11
	150	950	1.75	29.1	0.56	9.69E3	1.71E4	3.09	5.84E-13
	170	948	2.84	33.9	1.04	4.60E3	1.39E4	2.48	3.32E-13
	190	940	4.06	37.8	1.66	2.41E3	1.12E4	2.12	4.61E-13
	210	929	5.44	43.4	2.51	1.29E3	1.12E4	1.81	3.62E-13
	230	911	6.49	49.0	3.34	777	1.35E4	1.73	3.87E-12
	250	890	7.20	55.7	4.12	509	2.52E4	1.68	3.93E-11
	270	868	7.56	62.3	4.71	390	2.19E4	1.60	1.72E-10

300	845	7.86	67.8	5.16	277	2.21E4	1.44	6.25E-10

V_{OC}, open-circuit voltage; J_{SC}, short-ciucuit current density; FF, fill factor; PCE, power conversion efficiency; R_s, series resistance; R_{sh}, shunt resistance; n, ideality factor; J_0, reverse saturation dark current density.

The following are experimental results of OSCs based on PTB7:$PC_{61}BM$ (for simplicity, we call it PTB7 device). Here to facilitate comparison with P3HT:PCBM system, $PC_{61}BM$ was chosen as the acceptor, the concentration of solution and process of spin coating also stay the same with that of P3HT:PCBM device. The J-V curves under light and in dark are shown in Figure S7, extracted parameters of PTB7 device are shown in Table S2. From which it can be seen that the behaviours of PTB7 device under low temperature are consistent with those of P3HT devices. We have applied SB model and TE theory to the PBT7 device, and found that they are also applicable in OSCs based on low band gap donor material.

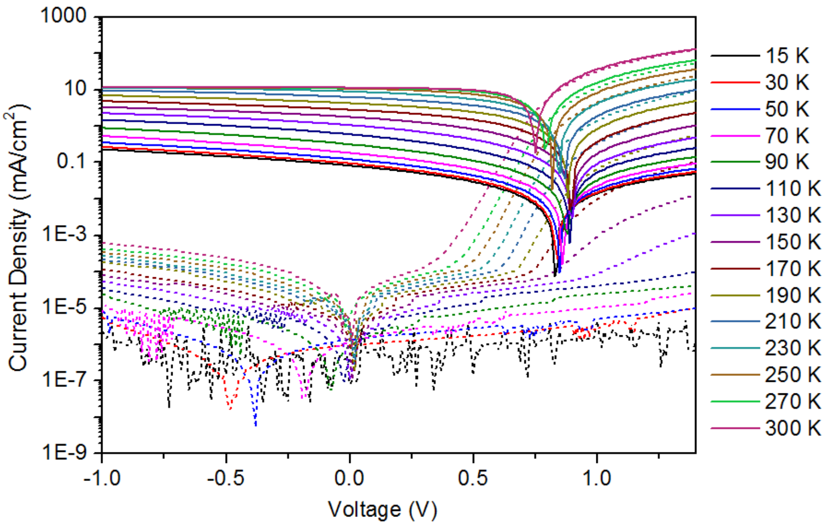

Figure S7 | Device performance under illumination and in dark. J-V characteristics measured in the temperature range of 15 - 300 K for PTB7:PCBM device.

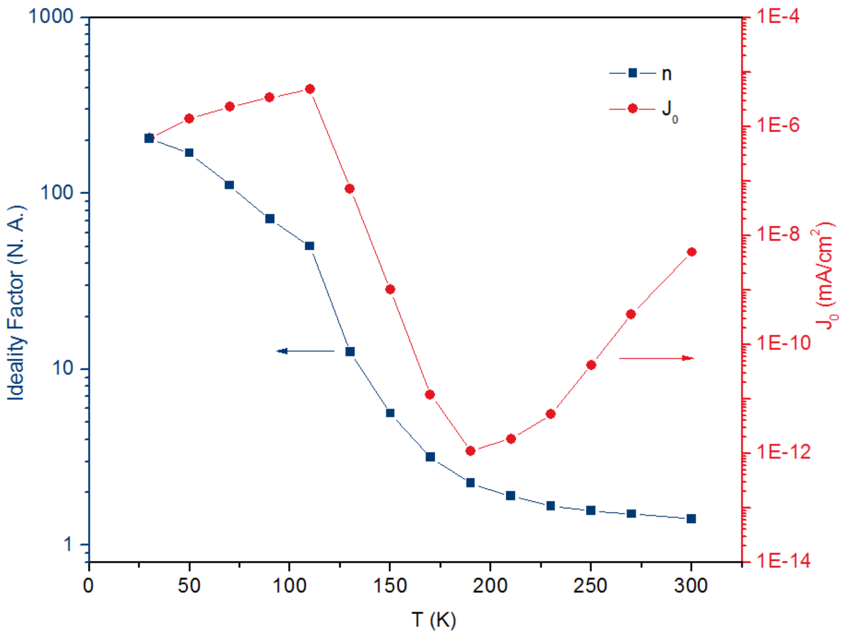

Figure S8 | n and J0 of PTB7:PCBM device.

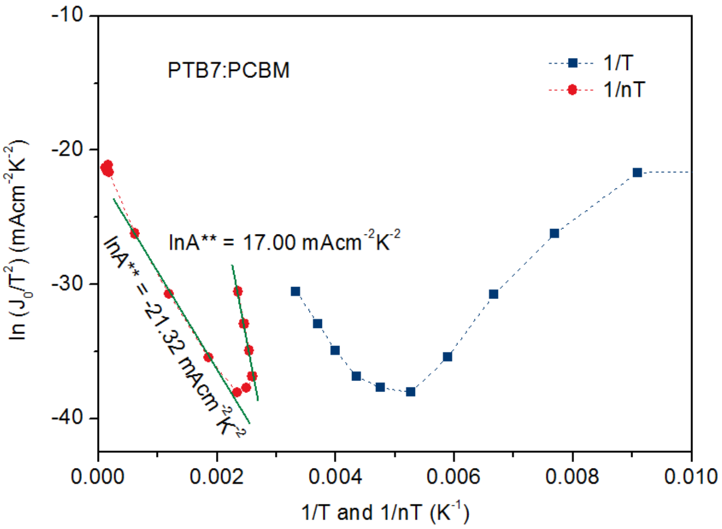

Figure S9 | $ln(J_0/T^2)$ vs 1/T and 1/nT. The plots of $ln(J_0/T^2)$ with 1/T and 1/nT for PTB7:PCBM device.

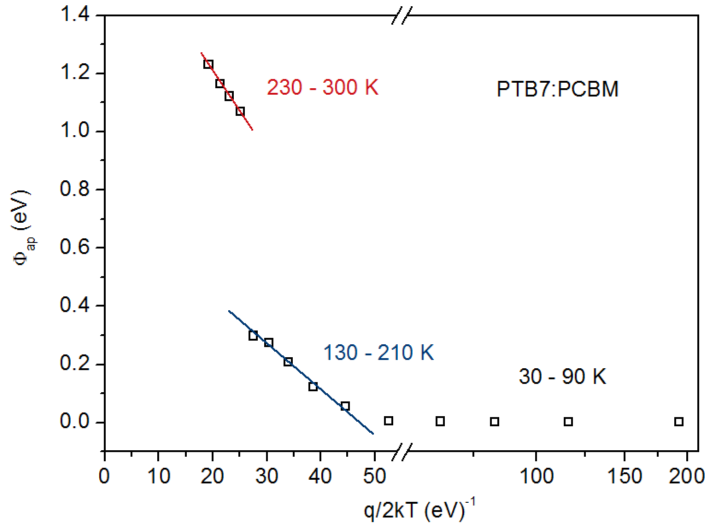

Figure S10 | Distribution of apparent SB height with Gaussian model. The apparent SB height plotted with q/2kT for PTB7:PCBM device.

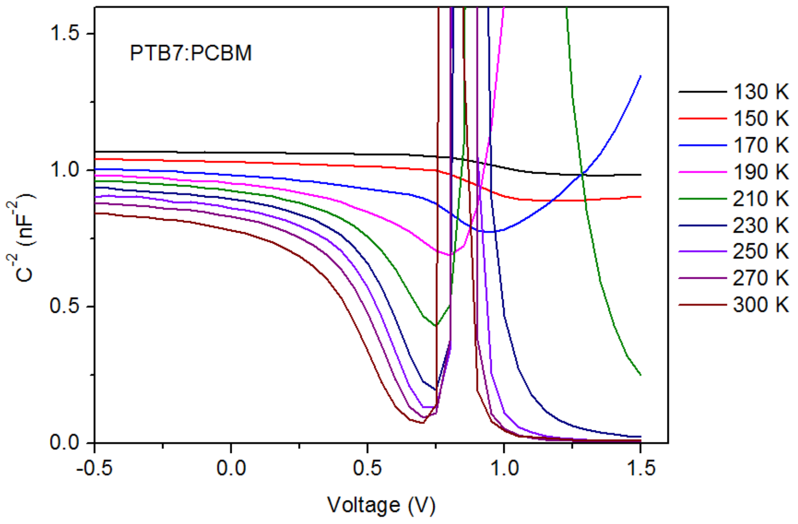

Figure S11 | Built-in potential. Mott-Schottky plots of PTB7:PCBM device under different temperatures.

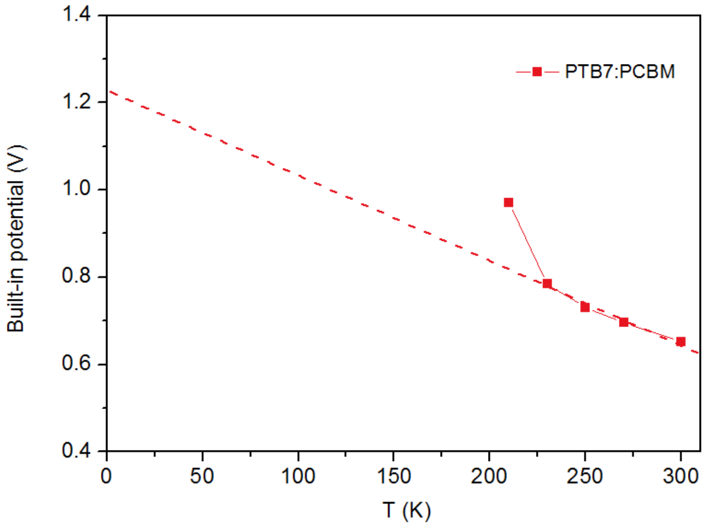

Figure S12 | Built-in potentials fitting. Built-in potentials obtained by C-V measurements for PTB7:PCBM device under different temperatures.

Table S2 | Device performance and extracted parameters of PTB7:PCBM device under different temperatures.

T [K]	V_{OC} [mV]	J_{SC} [mA/cm^2]	FF [%]	PCE [%]	R_s [Ω]	R_{sh} [Ω]	n [n.a.]	J_0 [mA/cm^2]
15	831	8.14E-2	23.0	1.94E-2	2.51E5	1.97E5	--	--
30	836	9.32E-2	22.8	2.22E-2	2.21E5	1.66E5	204	6.01E-7
50	847	0.122	22.4	2.89E-2	1.74E5	1.26E5	170	1.41E-6
70	861	0.182	22.0	4.30E-2	1.24E5	8.24E4	111	2.32E-6
90	876	0.313	21.4	7.34E-2	7.97E4	4.78E4	71.4	3.36E-6
110	889	0.587	21.6	0.141	4.19E4	2.77E4	50.2	4.74E-6
130	896	1.053	23.3	0.274	2.03E4	1.84E4	12.5	7.04E-8
150	896	1.760	26.2	0.516	8.79E3	1.33E4	5.62	1.03E-9
170	891	2.785	29.8	0.924	4.07E3	9.99E3	3.16	1.18E-11
190	881	4.201	32.6	1.51	2.06E3	7.46E3	2.25	1.10E-12
210	864	6.175	34.3	2.29	1.08E3	5.30E3	1.91	1.86E-12
230	842	8.807	36.6	3.39	572	4.66E3	1.68	5.23E-12
250	820	10.48	43.0	4.62	301	8.39E3	1.57	4.20E-11
270	794	11.03	51.3	5.62	166	1.35E4	1.51	3.61E-10
300	752	11.27	58.4	6.18	85	1.74E4	1.41	4.92E-9

V_{OC}, open-circuit voltage; J_{SC}, short-ciucuit current density; FF, fill factor; PCE, power conversion efficiency; R_s, series resistance; R_{sh}, shunt resistance; n, ideality factor; J_0, reverse saturation dark current density.
